# Temperature responsive chromatography for therapeutic drug monitoring with an aqueous mobile phase

**DOI:** 10.1038/s41598-021-02998-2

**Published:** 2021-12-06

**Authors:** Kenichi Nagase, Teruno Nishiyama, Masakazu Inoue, Hideko Kanazawa

**Affiliations:** Faculty of Pharmacy, Keio University, 1-5-30 Shibakoen, Minato, Tokyo 105-8512 Japan

**Keywords:** Analytical chemistry, Green chemistry

## Abstract

Therapeutic drug monitoring is a key technology for effective pharmacological treatment. In the present study, a temperature-responsive chromatography column was developed for safe and simple therapeutic drug monitoring without the use of organic solvents. Poly(*N*-isopropylacrylamide) (PNIPAAm) hydrogel-modified silica beads were prepared via a condensation reaction and radical polymerization. The temperature-dependent elution behavior of the drugs was observed using a PNIPAAm-modified silica-bead packed column and an all-aqueous mobile phase. Sharp peaks with reproducible retention times were observed at temperatures of 30 °C or 40 °C because the PNIPAAm hydrogel on the silica beads shrinks at these temperatures, limiting drug diffusion into the PNIPAAm hydrogel layer. The elution behavior of the sample from the prepared column was examined using a mixture of serum and model drugs. The serum and drugs were separated on the column at 30 °C or 40 °C, and the concentration of the eluted drug was obtained using the calibration curve. The results show that the prepared chromatography column would be useful for therapeutic drug monitoring because the drug concentration in serum can be measured without using organic solvents in the mobile phase and without any need for sample preparation.

## Introduction

In pharmacological therapy, the optimal drug dosage is different among patients because of the individual variation in adsorption, distribution, and elimination^1^. The dosage of particular types of drugs must be adjusted based on monitoring the drug concentration in serum because a drug at a low concentration is ineffective and an excessively high drug concentration can cause toxicity. Therapeutic drug monitoring that measures drug concentration in serum is an effective approach to monitor the therapeutic levels required for specific types of drugs^2,3^. Various measurement methods have been developed to determine drug concentration in serum including immunological and chromatographic methods^4^. Liquid chromatography is effective for determining drug concentration in serum because of its versatility and lack of requirement of a specific ligand for a particular drug^5^. However, liquid chromatography requires the use of the organic-solvent mobile phase to move drugs along the chromatography column. In addition, the blood sample must be deproteinated using organic solvents before measuring the drug concentration in the serum by chromatography. The use of organic solvents is often undesirable in hospitals because of the associated hazards of exposure. Thus, an analytical method that does not use organic solvents is required for therapeutic drug monitoring. Temperature-responsive chromatography using poly(*N*-isopropylacrylamide) (PNIPAAm) is one such measurement method that does not use organic solvents. PNIPAAm exhibits temperature-dependent hydrophilic and hydrophobic changes across its phase transition temperature of 32°C^6–9^. Since the phase transition temperature is close to the body temperature, PNIPAAm has been used in various biomedical applications such as temperature-modulated drug and gene delivery systems^10–15^, biosensors and bioimaging systems utilizing the phase transition properties of PNIPAAm^16–21^, cell separation systems using the difference in cell adhesive properties among cells on PNIPAAm^22–30^, and cell culture substrates for tissue engineering using temperature-modulated cell adhesion and detachment^31–38^. When applied to chromatography, PNIPAAm-modified silica beads are used as the stationary phase^39–44^. Because the hydrophobicity of PNIPAAm changes according to the external temperature, the hydrophobic interaction between PNIPAAm and the analyte can be modulated by changing the column temperature. The retention time of the analyte is modulated by changing the column temperature without changing the mobile phase composition; aqueous mobile phases can be used, thus alleviating the need for organic solvents.

**Figure 1:**
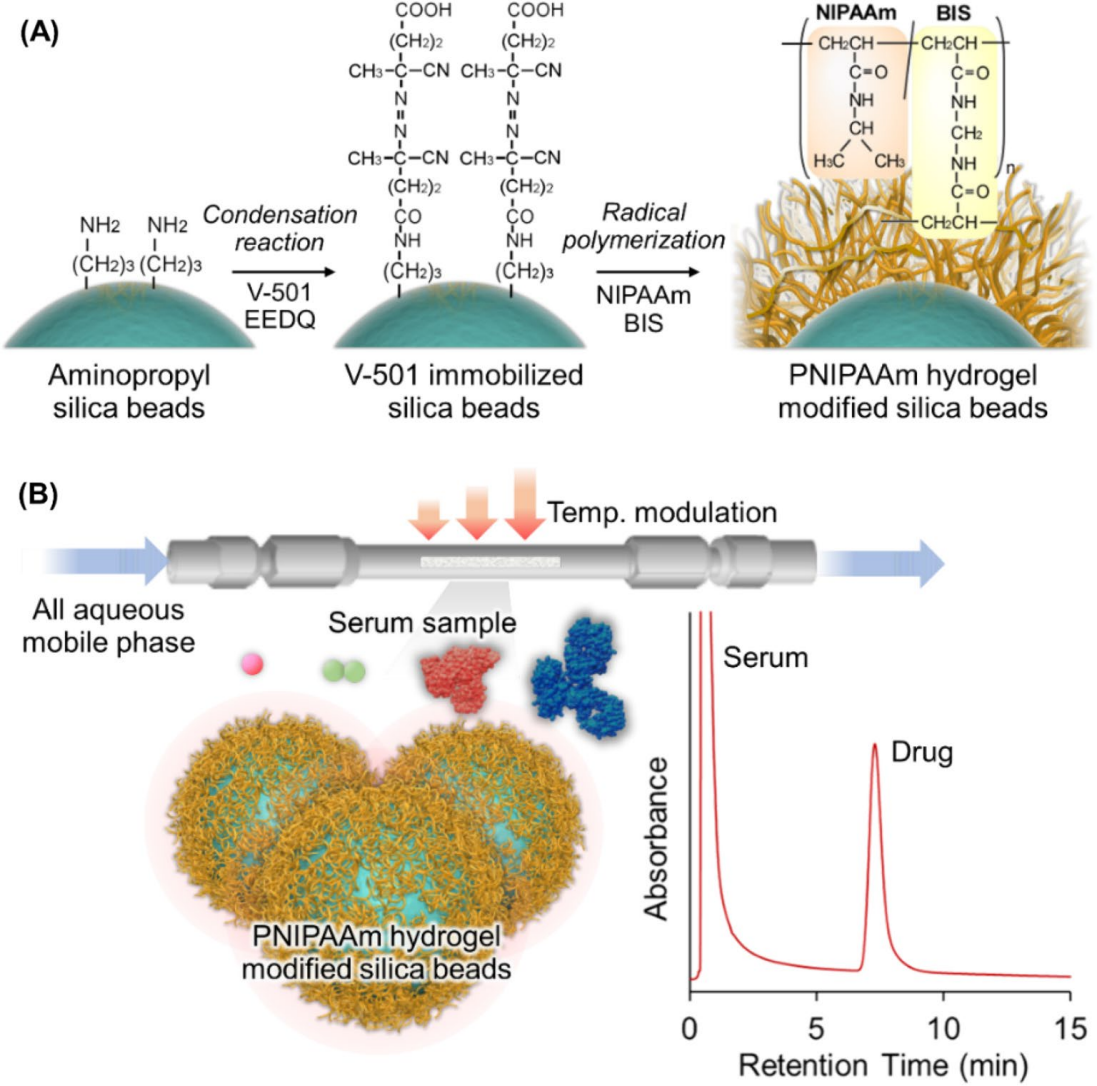
Schematic illustration of the preparation of thermoresponsive polymer hydrogel modified beads (**A**) and therapeutic drug monitoring using temperature-responsive chromatography with all-aqueous mobile phase (**B**).

In the present study, we developed PNIPAAm-modified silica beads as temperature-responsive chromatographic matrices for therapeutic drug monitoring (Fig. 1). Ten different drugs that required therapeutic drug monitoring were used as the analytes. The drug retention profiles were observed at various temperatures. Drugs and serum were separated using the PNIPAAm-modified silica bead column to investigate the viability of the temperature-responsive chromatography column for therapeutic drug monitoring.

## Results and discussion

### Characterization of the prepared beads

PNIPAAm-hydrogel-modified silica beads were prepared through a 4,4-azobis(4-cyanovaleric acid) (V-501) modification of silica beads, followed by radical polymerization of NIPAAm and *N,N*-methylenebisacrylamide (BIS) (Fig. 1A). The prepared beads were characterized by CHN elemental analysis, attenuated total reflection Fourier-transform infrared spectroscopy (ATR/FT-IR), and scanning electron microscopy (SEM).

The silica beads were characterized at each reaction step by elemental analysis (Table 1). A higher carbon composition was observed in the V-501 immobilized beads compared to that of the aminopropyl silica beads, indicating that V-501 was successfully placed on the beads. The density of the V-501 on the bead surface was 1.44 μmol/m^2^. This value is similar to that reported previously^45,46^. The PNIPAAm-hydrogel-modified beads exhibited a higher carbon composition compared to that of the V-501-immobilized silica beads. The results indicated that the PNIPAAm hydrogel was modified on the silica beads via polymerization of NIPAAm and BIS. PNIPAAm-modified silica beads prepared via either 5 h or 18 h polymerization exhibited different amounts of PNIPAAm, 0.098 mg/m^2^ or 0.215 mg/m^2^, respectively. A longer polymerization reaction resulted in a larger amount of PNIPAAm on the silica beads. This also indicated that the larger molecular weight of PNIPAAm was present on silica beads prepared by polymerization for 18 h compared to 5 h.

**Table 1:** Characterization of thermo-responsive hydrogel-modified beads using CHN elemental analysis.

Beads	Elemental composition (%)		%C_(calcd)_	Immobilized initiator (μmol/m^2^)	Grafted polymer (mg/m^2^)
	Carbon	Nitrogen			
Aminopropyl silica beads	2.98 ± 0.01	1.10 ± 0.01			
V-501 immobilized silica beads	8.69 ± 0.06	2.49 ± 0.03	51.4	1.44	
PNIPAAm hydrogel modified beads (5 h polymerization)	10.36 ± 0.06	2.49 ± 0.03	63.7		0.098
PNIPAAm hydrogel modified beads (18 h polymerization)	12.23 ± 0.03	2.80 ± 0.03	63.7		0.216

%C_(calcd)_ was calculated as the percentage of the molecular weight of carbon in the initiator and polymer. The amount of initiator and polymer on silica beads were estimated using the carbon composition.

**Figure 2:**
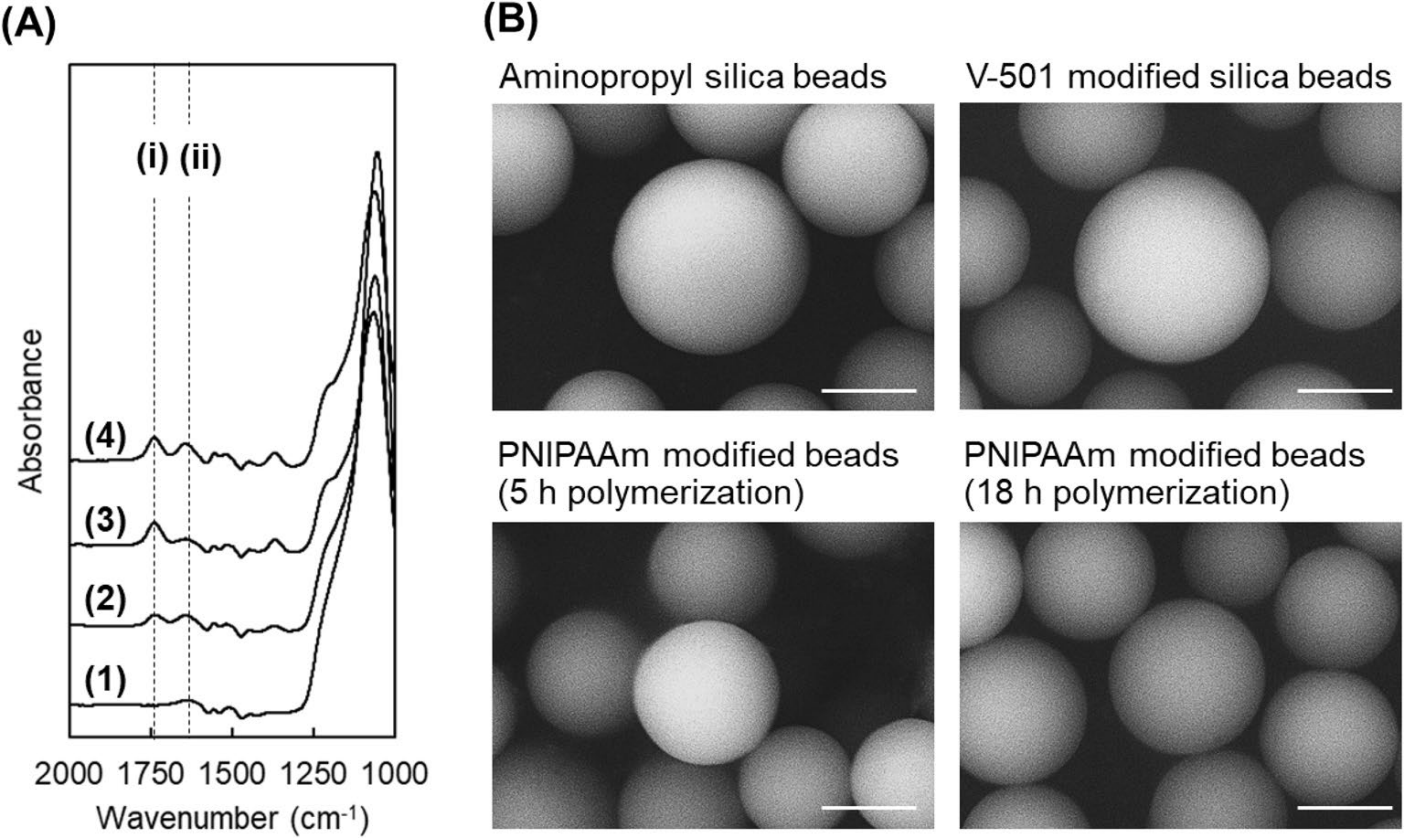
Characterization of the prepared beads. (**A**) Fourier transfer infrared spectra of the prepared beads. The dashed lines (i) and (ii) indicate the peak attributed to C=O stretching vibrations and N–H bending vibrations, respectively. (**B**) SEM images of the prepared beads. (1) aminopropyl silica beads, (2) V-501 immobilized silica beads, (3) PNIPAAm modified silica beads prepared via polymerization for 5 h, and (4) PNIPAAm modified silica beads prepared via polymerization for 18 h. Scale bars denote 5 μm.

The prepared beads were characterized by ATR/FT-IR at each reaction step (Fig. 2A). Two peaks at 1550 and 1645 cm^−1^ were observed in the spectra of the PNIPAAm-modified beads, while no peaks were observed in the spectra of the aminopropyl silica beads. The peaks were attributed to the N–H bending vibration and C=O stretching vibrations of the N–H and C=O bonds, respectively, of the modified PNIPAAm hydrogel on the silica beads. Thus, the results further confirmed that PNIPAAm was successfully generated on silica beads via a polymerization reaction.

The morphology of the prepared beads was observed at each step using scanning electron microscopy (SEM) (Fig. 2B). A similar spherical shape was observed after the V-501 immobilization and PNIPAAm polymerization reaction steps. These results suggest that the V-501 immobilization reaction and PNIPAAm modification reaction did not deform the silica beads. Additionally, aggregated beads were not observed after polymerization, indicating that the polymerization was relatively controlled during both reaction times.

### Evaluation of column performance

The prepared PNIPAAm-hydrogel-modified beads were packed into a stainless-steel column (4.6 mm internal diameter × 50 mm length).

**Figure 3:**
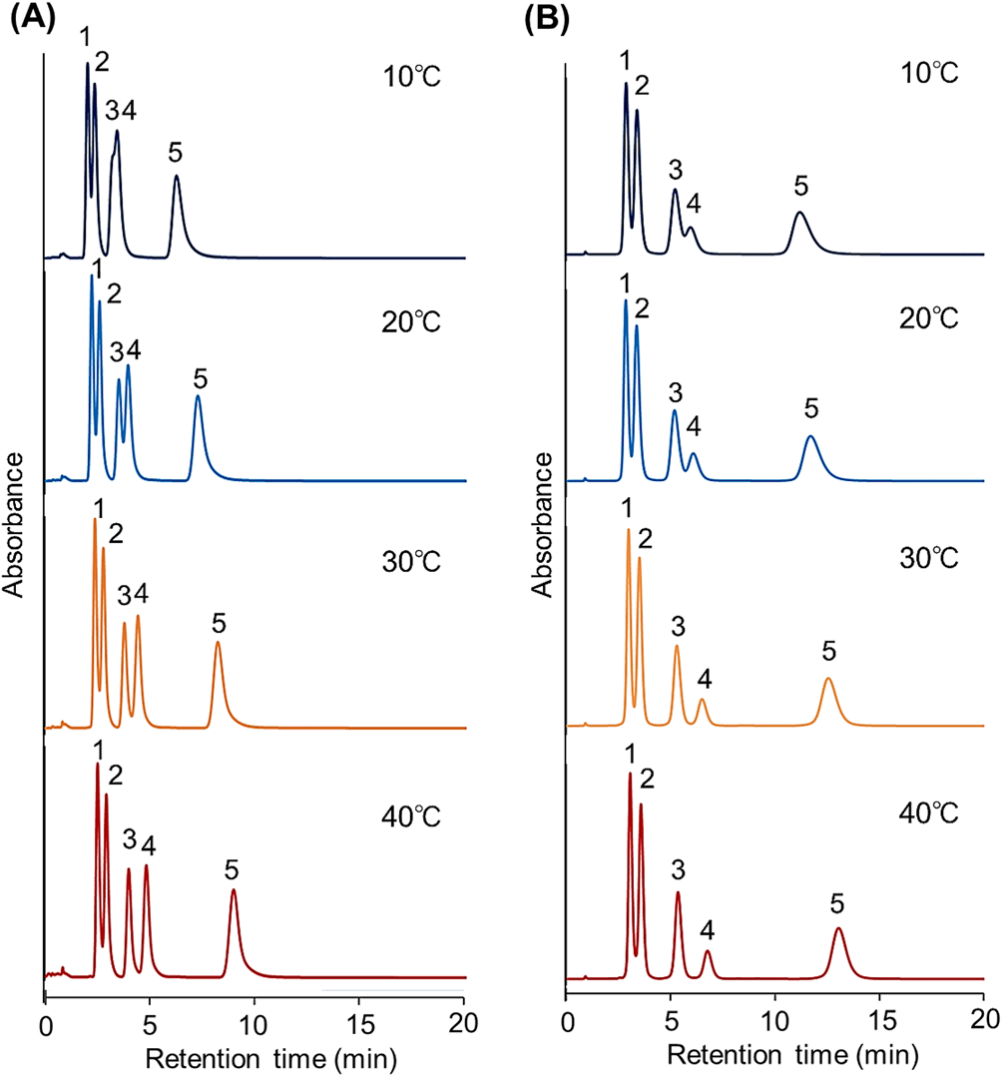
Chromatograms of hydrophobic steroids using crosslinked PNIPAAm modified-bead packed columns using beads prepared via polymerization for (**A**) 5 h, and (**B**) 18 h. The mobile phase was pure water. The flow rate of the mobile phase was 1.0 mL/min. Detection at 254 nm. Peak 1 = hydrocortisone, peak 2 = prednisolone, peak 3 = dexamethasone, peak 4 = hydrocortisone acetate, and peak 5 = testosterone.

To investigate the column performance of the prepared beads, the elution behavior of five hydrophobic steroids—hydrocortisone, prednisolone, dexamethasone, hydrocortisone acetate, and testosterone, was observed (Fig. 3). The properties of the steroids are summarized in Table S1. The retention time of the steroids increased with increasing column temperature on the columns prepared via the two polymerization reaction times of 5 h and 18 h. This is because the hydrophobic interaction between PNIPAAm and the steroids increased with increasing temperature. The PNIPAAm hydrogel dehydrated and became hydrophobic with increasing temperature, leading to enhanced hydrophobic interactions between the PNIPAAm and the steroids, and thus an elongation of the retention time. A longer retention time was observed on the column made using the beads prepared via the 18 h polymerization compared with that made using the beads prepared via the 5 h polymerization. This is because the hydrophobic interaction between PNIPAAm and the steroids increased with the increasing amount of PNIPAAm on the silica beads, that is, with an increased molecular weight of the PNIPAAm hydrogel on silica beads^47,48^. However, wider peaks were observed on the column using the beads prepared via the 18 h polymerization compared to that using the beads prepared via the 5 h polymerization because of the longer diffusion time of the steroid molecules into the thicker PNIPAAm hydrogel layer. The PNIPAAm hydrogel layer coated on the beads during the 18 h polymerization was thicker than that coated on the beads during the 5 h polymerization. Thus, the diffusion of the steroids into the thicker PNIPAAm hydrogel layer resulted in a slower elution from the beads and wider peaks in the chromatogram. Previous studies have suggested that small molecular analytes tend to diffuse into a thick PNIPAAm layer composed of large molecular weight PNIPAAm on silica beads at a higher temperature^44,47–50^.

Therefore, the column incorporating the beads prepared via the 5 h polymerization was used for these investigations because of the shorter retention times and narrower peaks in the chromatogram. A short analysis time is required for therapeutic drug monitoring because the serum drug concentration of patients should be measured promptly. In addition, wide peaks in the chromatograms reduced the accuracy of the determination.

### Elution behavior of drugs in therapeutic drug monitoring

The suitability of the prepared column was investigated for therapeutic drug monitoring using ten types of drugs that required therapeutic drug monitoring in therapy (Table S2).

**Figure 4:**
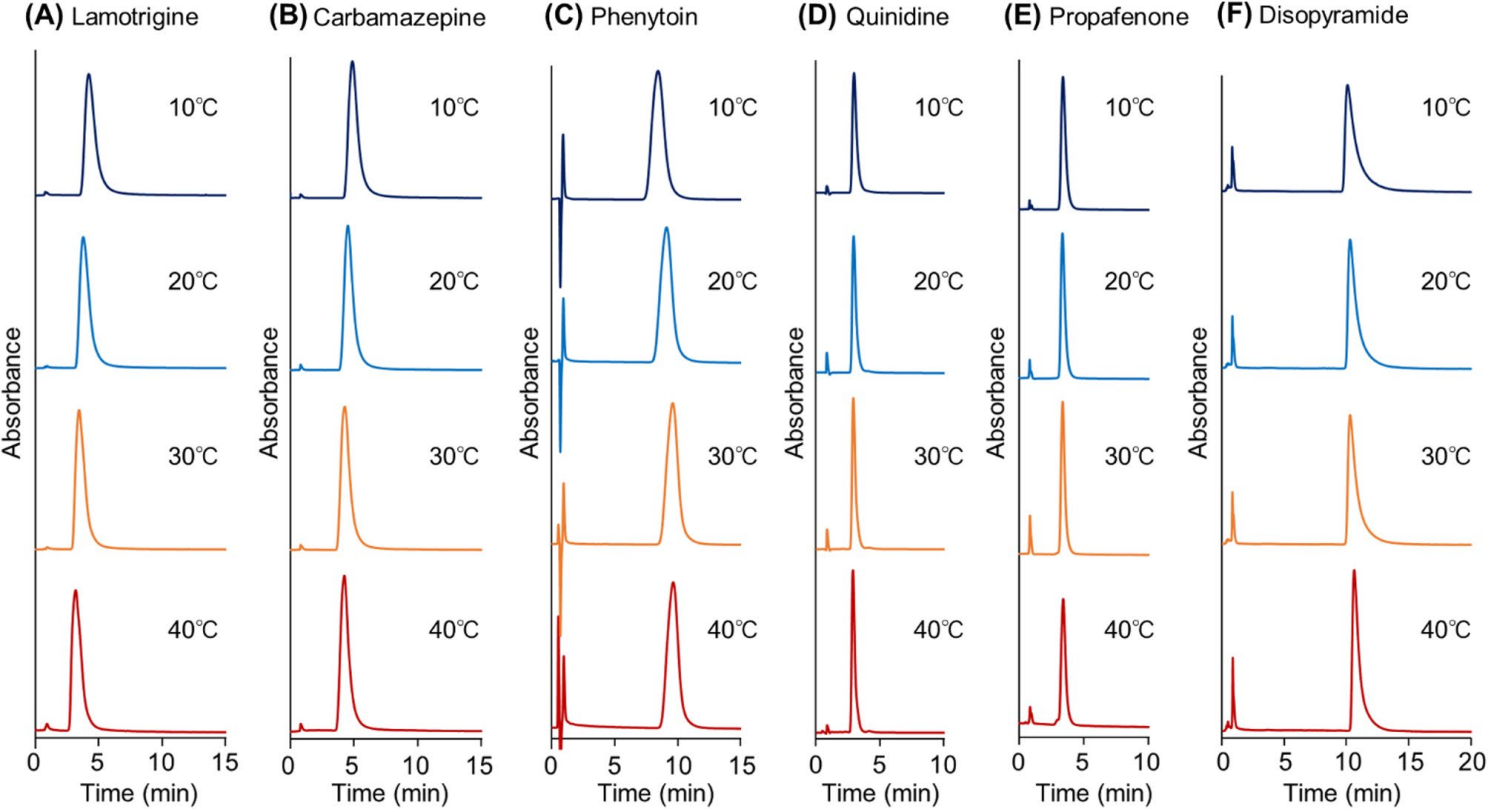
Chromatograms of antiepileptic drugs (**A**–**C**) and anti-arrhythmic drugs (**D**–**F**) using crosslinked PNIPAAm modified-bead packed columns. (**A**) Lamotrigine, (**B**) Carbamazepine, (**C**) Phenytoin, (**D**) Quinidine, (**E**) Propafenone, and (**F**) Disopyramide. The mobile phase was 10 mM CH_3_COONH_4_ (pH 6.75) buffer solution with a flow rate of 1.0 mL/min.

The elution behavior of antiepileptic drugs, lamotrigine, carbamazepine, and phenytoin was observed at various temperatures using 10 mM CH_3_COONH_4_ (pH 6.70) as the mobile phase (Fig. 4A–C). The retention time of phenytoin increased with increasing column temperature because the hydrophobic interactions increased with increasing temperature, similar to the tendency exhibited by the hydrophobic steroids. In contrast, the retention times of lamotrigine and carbamazepine were shorter than those of phenytoin, probably because phenytoin contains N–H and C=O bonds that contribute to the formation of hydrogen bonds with PNIPAAm. For all three drugs, the peak width decreased with increasing temperature because the analyte tended to diffuse into the swollen PNIPAAm hydrogel layer at low temperatures, leading to slower elution from the beads and wider peaks in the chromatogram. However, at high temperatures, the PNIPAAm hydrogel layer shrank, resulting in a decreased extent of analyte diffusion.

The elution behavior of anti-arrhythmic drugs, quinidine, propafenone, and disopyramide was observed in the prepared column (Fig. 4D–F). Disopyramide exhibited a longer retention time than quinidine and propafenone, probably because the amide group of disopyramide interacts with PNIPAAm through hydrogen bonding. Disopyramide exhibited a wider peak at low temperatures because it diffused into the swollen PNIPAAm hydrogel layer and interacted with PNIPAAm at low temperatures. However, disopyramide exhibited a sharp peak at a higher column temperature (Fig. 4F) because the PNIPAAm hydrogel shrank resulting in decreased disopyramide diffusion. Quinidine and propafenone exhibited sharp peaks at both column temperatures because, unlike disopyramide, these analytes were retained only by hydrophobic interactions with the PNIPAAm hydrogel. Thus, the interaction between these analytes and PNIPAAm in the swollen PNIPAAm hydrogel layer was relatively weak compared to that of disopyramide, leading to narrower peaks.

**Figure 5:**
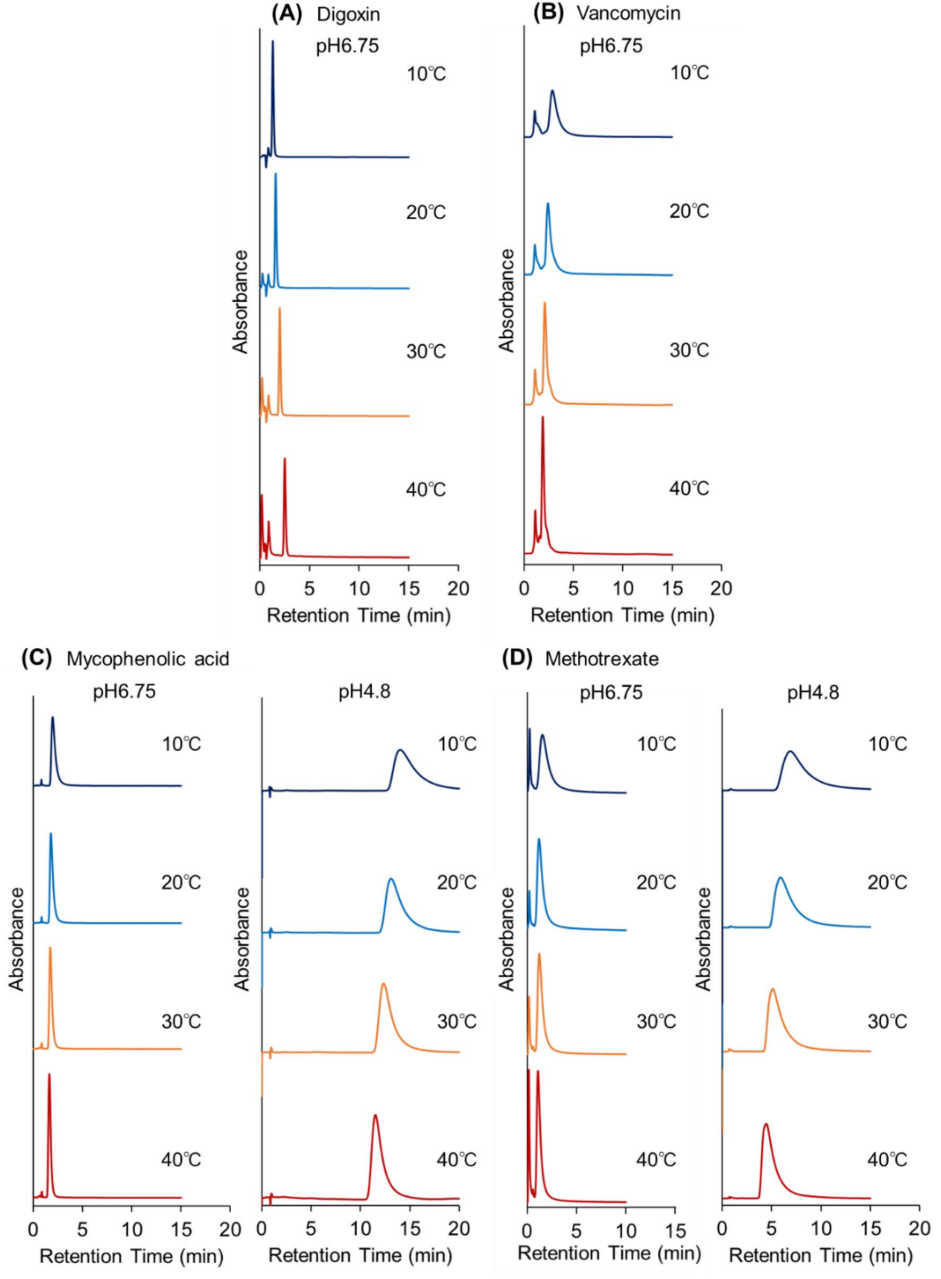
Chromatograms of (**A**) digoxin (cardiac glycoside), (**B**) vancomycin (antibacterial drug), (**C**) mycophenolic acid (immune-suppressing drug), and (**D**) methotrexate (anti-cancer drug) using crosslinked PNIPAAm modified-bead packed columns. Mobile phases were 10 mM CH_3_COONH_4_ (pH 6.75) buffer solution and 10 mM CH_3_COONH_4_ (pH 4.8) buffer solution with a flow rate of 1.0 mL/min.

The elution behavior of digoxin (a cardiac glycoside) and vancomycin (an antibacterial drug) in the prepared column is shown in (Fig. 5A, B). Digoxin exhibited a sharp elution peak in the chromatogram at a short retention time. A slight increase in the retention time of digoxin was observed with increasing temperature because the hydrophobic interaction between digoxin and PNIPAAm increased with increasing temperature. The chromatogram of vancomycin exhibited two peaks; the smaller peak at shorter retention time was attributed to a related substance of vancomycin.

The chromatograms of mycophenolic acid (an immune-suppressing drug) and methotrexate (an anti-cancer drug) are shown in Fig. 5C, D. The retention of mycophenolic acid using a pH 6.75 buffer solution was short, and a sharp peak was observed (Fig. 5C). Thus, the pH of the mobile phase was changed to 4.80 to suppress the dissociation of the carboxyl group in mycophenolic acid and to increase the hydrophobicity of mycophenolic acid. The retention time of the mycophenolic acid increased. Methotrexate was also eluted within a short retention time when pH 6.75 buffer solution was used as the mobile phase (Fig. 5D). Thus, the pH of the mobile phase was changed to 4.80 to suppress the dissociation of the carboxyl groups in methotrexate and increase the hydrophobicity of methotrexate. In consequence, the retention time of methotrexate was increased when a pH 4.80 buffer was used as the mobile phase; this was again attributed to the enhanced hydrophobic interaction between PNIPAAm and methotrexate.

**Figure 6:**
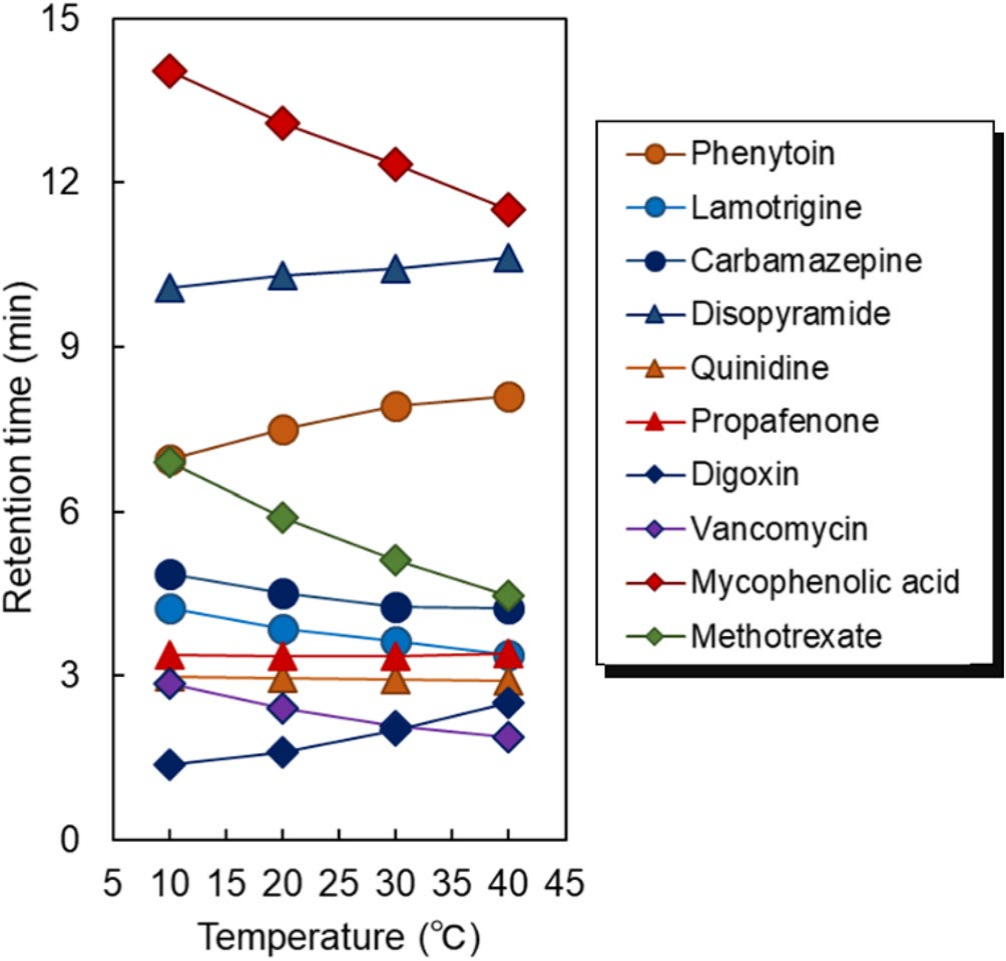
The retention time of drugs used in therapeutic drug monitoring using PNIPAAm hydrogel modified-bead columns. Retention times of mycophenolic acid and methotrexate were obtained using pH 4.80 CH_3_COONH_4_ buffer solution as mobile phase. All other drugs used pH 6.75 CH_3_COONH_4_ buffer solution mobile phase.

The temperature-dependent retention time changes of all the analytes were summarized to compare the retention profiles with changing column temperature (Fig. 6). The retention times of mycophenolic acid and methotrexate were obtained using a pH 4.8 CH_3_COONH_4_ buffer solution as the mobile phase. A pH 6.75 CH_3_COONH_4_ buffer solution was used as the mobile phase for the other drugs. Most drugs could be analyzed within a short analysis time (8 min), although mycophenolic acid and disopyramide exhibited a slightly longer retention time. Various changes in the retention time with changing column temperature were observed. The retention times of disopyramide, phenytoin, and digoxin increased, whereas those of mycophenolic acid, methotrexate, and vancomycin decreased with increasing temperature. The retention times of the other drugs, carbamazepine, lamotrigine, propafenone, and quinidine were almost independent of the column temperature. The difference in retention time with temperature is attributed to the differences in the analyte properties such as hydrophobicity and ionic properties. PNIPAAm on the column silica beads becomes hydrophobic with increasing temperature due to dehydration. Thus, the hydrophobic interaction between PNIPAAm and the analyte increase, leading to an increase in the retention time of these analytes. In contrast, the solubility of the analytes in the mobile phase increase with increasing temperature, leading to a shortening of the retention time of the analytes. The retention of the analyte is dependent on the balance between these two factors. In the case of disopyramide, phenytoin, and digoxin, the effect of increasing the hydrophobic interaction between PNIPAAm and the analyte with increasing temperature was large compared to the effect of increasing analyte solubility in the mobile phase. In contrast, for mycophenolic acid, methotrexate, and vancomycin, the effect of increasing analyte solubility in the mobile phase was large compared to the effect of increasing hydrophobic interactions between PNIPAAm and the analyte. In the case of carbamazepine, lamotrigine, propafenone, and quinidine, both effects were balanced at all temperatures, leading to the retention time being independent of temperature.

The reproducibility was investigated by observing three separate measurements of the retention time and expressed as the RSD value (Table S3). The RSD of all the measurements was low, indicating that the prepared column provided reproducible data.

### Separation of drugs and serum for therapeutic drug monitoring

**Figure 7:**
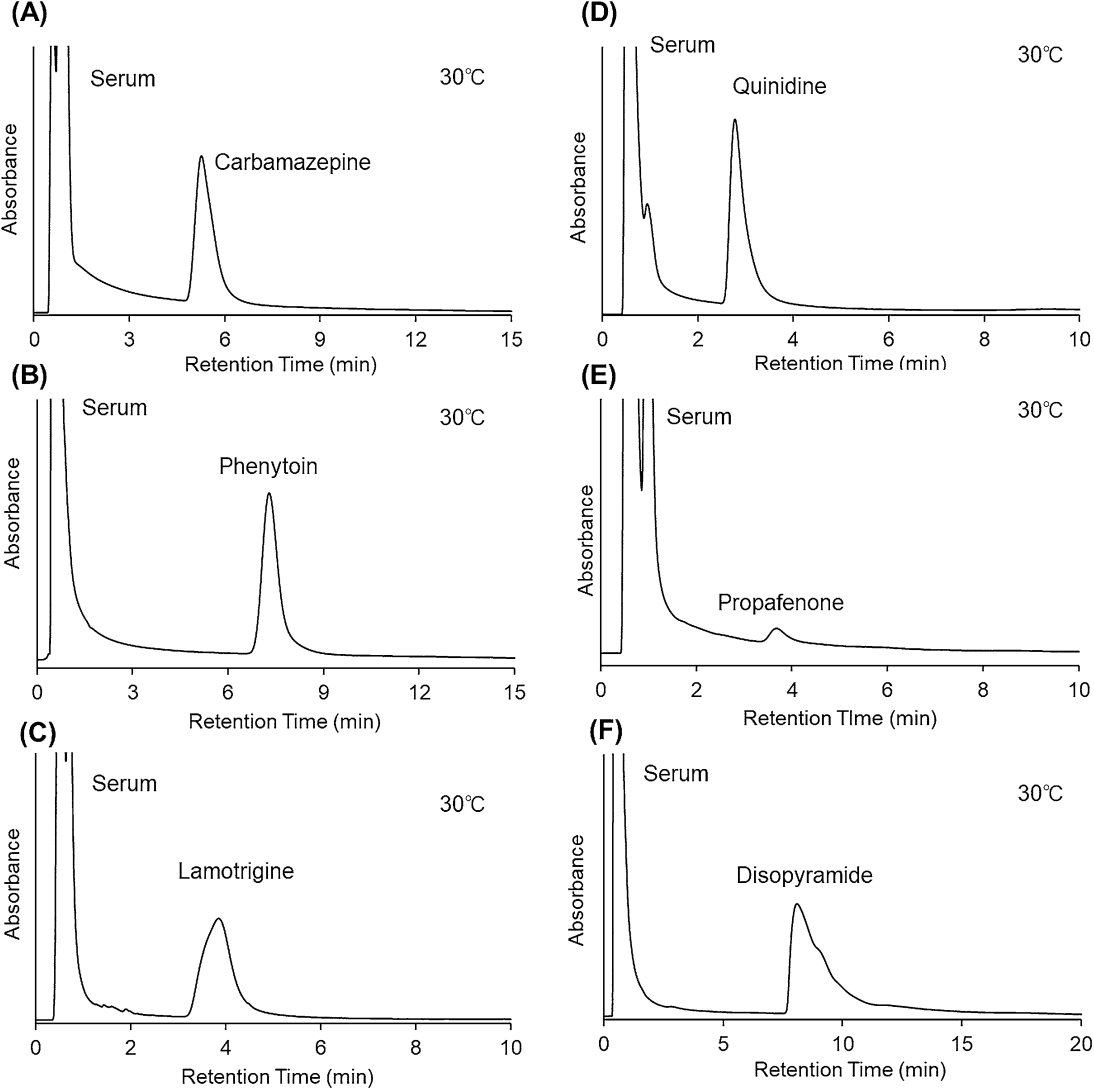
Chromatograms of antiepileptic drug and anti-arrhythmic drug with serum proteins using crosslinked PNIPAAm modified-bead packed columns. (**A**) Carbamazepine, (**B**) phenytoin, (**C**) lamotrigine, (**D**) quinidine, (**E**) propafenone, and (**F**) disopyramide. Mobile phase was 10 mM CH_3_COONH_4_ (pH 6.75) buffer solution for carbamazepine, phenytoin, lamotrigine, and disopyramide, and 10 mM CH_3_COONH_4_ buffer solution (pH 4.80) for quinidine and propafenone. The mobile phase flow rate was 1.0 mL/min.

**Figure 8:**
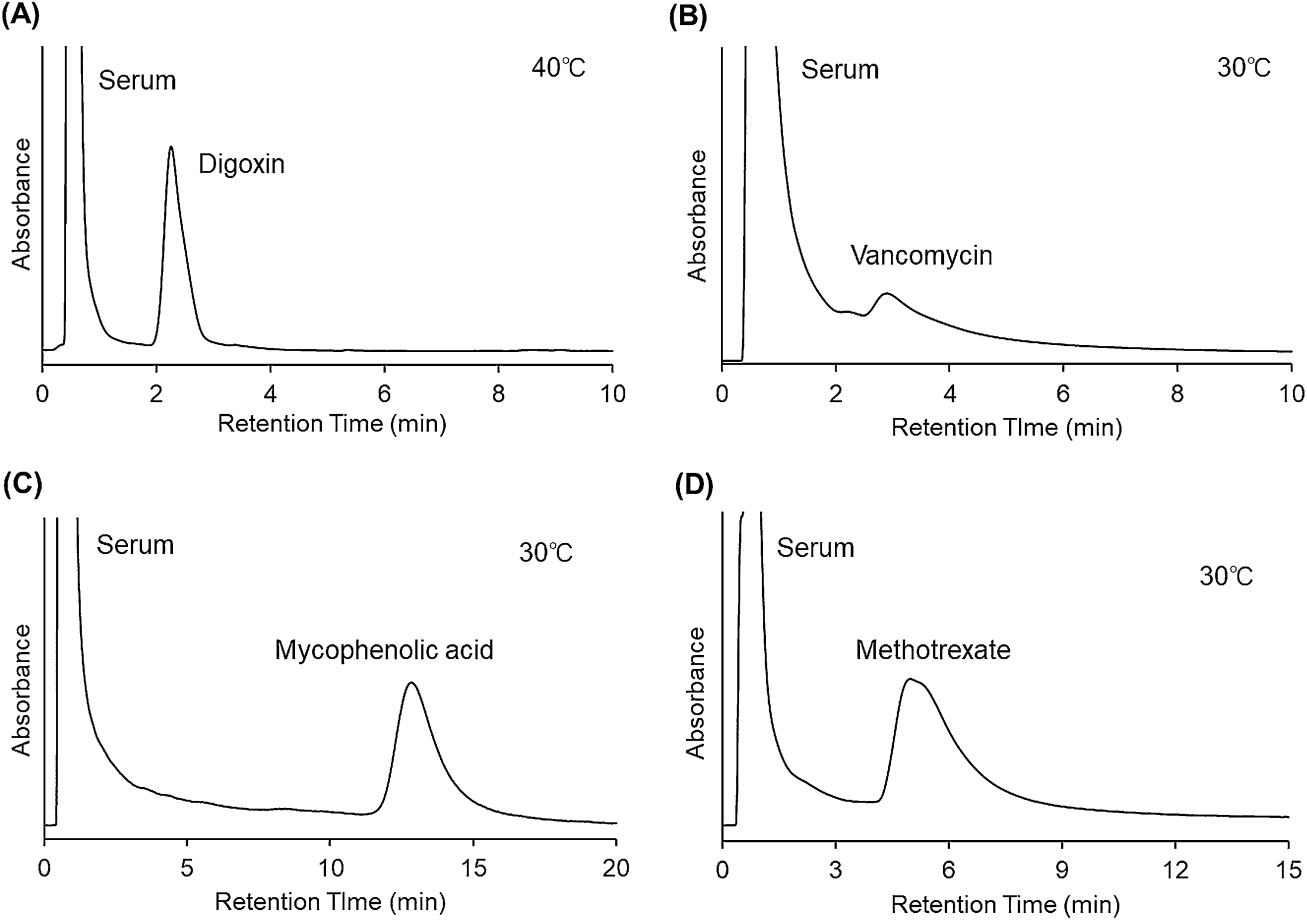
Chromatograms of (**A**) digoxin, (**B**) vancomycin, (**C**) mycophenolic acid, and (**D**) methotrexate in serum using crosslinked PNIPAAm modified-bead packed columns. The mobile phase was 10 mM CH_3_COONH_4_ buffer solution (pH 6.75) for digoxin, and 10 mM CH_3_COONH_4_ buffer solution (pH 4.80) for vancomycin, mycophenolic acid, and methotrexate. The mobile phase flow rate was 1.0 mL/min.

Ordinarily, drug concentration must be determined in serum, and deproteination of the serum sample (by the addition of organic solvent) is required before HPLC column injection to avoid serum protein adsorption onto the beads in the column resulting in the column clogging. Thermoresponsive ionic copolymers composed of NIPAAm and ionic monomers have been shown to adsorb proteins via multiple hydrophobic and electrostatic interactions^51–58^. In contrast, the PNIPAAm homopolymer modified-bead packed column scarcely adsorbs proteins even at high column temperatures^51,52^. Thus, the PNIPAAm homopolymer column prepared in the present study could be used for serum samples without deproteination because serum proteins do not adsorb onto the column beads. The suitability of the prepared column for determining drug concentration in serum was investigated using a mixture of serum and model drugs (Figs. 7 and 8). The column temperature was modulated to an appropriate temperature to achieve separation of the drug from serum proteins and to obtain sharp peaks to increase quantitation accuracy.

The elution behavior of antiepileptic drugs in the serum was observed (Fig. 7A–C). The column temperature was set at 30 °C because of the sharp peaks and relatively longer retention time of phenytoin. The serum was eluted first followed by drugs’ elution; the serum was barely retained on the column compared to longer drugs retention; all the drugs were eluted within 9 min, a sufficiently short time for rapid therapeutic drug monitoring.

For the determination of anti-arrhythmic drugs in the serum (Fig. 7D–F), the column temperature was also set at 30 °C because of the sharp peaks of these drugs and the longer retention time of disopyramide. All three anti-arrhythmic drugs were separated from the serum proteins. Although a slightly longer elution time was observed for disopyramide, analysis was performed within an acceptable time.

The separation of four types of drugs, digoxin, vancomycin, mycophenolic acid, and methotrexate from serum was investigated at 30 °C (Fig. 8). The elution behavior of digoxin and serum was observed at 40 °C because the retention time of digoxin was short at 30 °C, with the separation of digoxin and serum not being adequate at this temperature. A pH 4.80 mobile phase was used for mycophenolic acid and methotrexate to increase their retention and separation from the serum. The serum and drugs were separated using the prepared column, although the peak of vancomycin overlapped slightly with that of serum.

There is a possibility for drug binding to serum proteins, and the bound drug being eluted with serum proteins. However, in a previous study, we investigated the effect of drug binding on serum proteins on separation using PNIPAAm-modified silica beads^46^; we reported that drug binding to serum proteins did not in this case affect separation between drug and serum proteins^46^.

The separated peaks from serum were observed for each of the types of drugs using the prepared column. Thus, the developed column can determine the drug concentration in serum using all aqueous mobile phases and without sample deproteination, whereas the conventional method using a reversed-phase chromatography system requires an organic mobile phase and deproteination using an organic eluent.

The calibration curves of seven drugs—phenytoin, lamotrigine, carbamazepine, disopyramide, quinidine, propafenone, mycophenolic acid, digoxin, and methotrexate in the effective drug concentration range for therapeutic drug monitoring were quantified using the method reported here (Fig. S1). The effective drug concentration range of digoxin and methotrexate could not be determined because the effective concentration ranges are small, 0.8–20 ng/mL, below the 2.27 μg/mL limit. A linear approximation was obtained between the drug concentration and the peak area of the obtained peak (Fig. S1). A high correlation coefficient was observed in each calibration curve (above 0.997), indicating that the drug concentration can be determined using the prepared column. The peak area at the lowest concentration of disopyramide (5 μg/mL) and mycophenolic acid (1 μg/mL) differed slightly from the linear approximation, leading to a relatively low correlation coefficient (disopyramide for 0.9982 and mycophenolic acid for 0.9973) compared to the other analytes. Thus, in the case of these two analytes, a more accurate correlation can be obtained above the lowest concentrations.

These results indicate that the drug concentration in serum proteins can be measured using the developed PNIPAAm-hydrogel-modified silica bead packed column with an all-aqueous mobile phase and without pre-deproteination of samples. The developed chromatography column is useful for therapeutic drug monitoring, and the chromatography system is safe in a medical setting because of the absence of organic solvents in the mobile phase; further, it does not require deproteination as a sample preparation step.

## Conclusions

A temperature-responsive chromatography column was developed for safe and simple therapeutic drug monitoring. PNIPAAm hydrogel-modified beads were prepared via the immobilization of V-501 on aminopropyl silica beads using a condensation reaction and subsequent radical polymerization with a polymerization time of either 5 h or 18 h. Silica beads prepared via the 18 h radical polymerization exhibited a larger concentration of PNIPAAm on the silica beads compared to silica beads prepared via the 5 h radical polymerization. Shorter retention times and sharper peaks were observed for the column using the beads prepared via the 5 h polymerization compared to using the beads prepared via the 18 h polymerization because a thinner PNIPAAm hydrogel layer was deposited on the silica beads via the 5 h polymerization and the thin PNIPAAm hydrogel layer reduced analyte diffusion into the PNIPAAm layer and hence the retention time. The elution behavior of ten types of drugs was examined to determine the feasibility of using the silica-bead packed column for therapeutic drug monitoring. All the drugs were retained on the prepared column and exhibited sharp peaks at high temperatures because the PNIPAAm hydrogel shrunk and prevented analyte diffusion into the hydrogel layer. The elution behavior from the column was determined using a mixture of serum and drugs as model samples. At 30 °C or 40 °C, the serum and drugs were separated, and the drug concentration was estimated using the calibration curve. The results indicated that the prepared temperature-responsive chromatography column would be useful for therapeutic drug monitoring because the chromatography column enables the determination of drug concentration in serum without using organic solvents in the mobile phase and without requiring sample deproteination.

## Methods

### Preparation of PNIPAAm hydrogel-modified beads

PNIPAAm hydrogel-modified silica beads were prepared using the following procedure (Fig. 1A). All materials used in the experiments are summarized in the Supplementary Information. 2-ethoxy-1-ethoxycarbonyl-1,2-dihydroquinoline (EEDQ) (6.18 g, 25.0 mmol) and 4,4-azobis(4-cyanovaleric acid) (V-501) (3.50 g, 12.5 mmol) were dissolved in *N*,*N*-dimethylformamide (DMF) (50 mL) in a flask. Aminopropyl silica beads (5.0 g) were added to the solution. The bead suspension was deoxygenated by bubbling with nitrogen gas for 30 min. The flask was sealed, and the condensation reaction was carried out for 6 h at 25 °C. After the reaction, the beads were filtered, rinsed with ethanol, and dried at 25 °C *in vacuo*.

NIPAAm (5.00 g, 44.2 mmol) and *N,N′*-methylene-*bis*acrylamide (BIS) (0.135 g, 0.876 mmol) were dissolved in ethanol (100 mL) in a flask. V-501 modified silica beads (2.0 g) were added to the solution. The bead suspension was deoxygenated for 30 min. The flask was then sealed, and radical polymerization proceeded with continuous shaking at 70 °C for either 5 or 18 h. Different amounts of PNIPAAm were generated on the bead surface by the different duration of polymerization. After the polymerization reaction, the beads were rinsed with methanol and dried at 25 °C *in vacuo*.

### Characterization of prepared beads

The prepared beads were characterized by CHN elemental analysis, FT-IR, and SEM at each reaction step.

The carbon composition of the prepared beads was measured using a CHN elemental analyzer (PE-2400, PerkinElmer, Waltham, MA, USA). The quantity of modified V-501 and PNIPAAm on the silica bead surfaces was obtained using the carbon composition of the beads (Eq. 1).

1 $$\frac{{\% C_{I} }}{{\% C_{I} \left( {calcd} \right) \times \left( {1 - \% C_{I} /\% C_{I} \left( {calcd} \right)} \right) \times S^{\prime } }}$$

where %*C*_*I*_ is the increase in carbon content after V-501 modification, %*C*_*I*_ (*calcd*) is the calculated carbon percentage of the V-501 molecule, and *S* is the surface area of the silica beads.

The quantity of PNIPAAm hydrogel on the silica bead surfaces was obtained using Eq. (2):

2 $$\frac{{\% C_{P} }}{{\% C_{P} \left( {calcd} \right) \times \left( {1 - \% C_{P} /\% C_{P} \left( {calcd} \right) - \% C_{I} /\% C_{I} \left( {calcd} \right)} \right) \times S^{\prime } }}$$

where %*C*_*p*_ is the increase in the carbon content of the PNIPAAm-modified beads relative to that of the V-501 modified beads, and %*C*_*P*_ (*calcd*) is the calculated carbon percentage of PNIPAAm.

Polymer modification of the silica beads surface was also confirmed by attenuated total reflection Fourier-transform infrared spectroscopy (FT/IR-4700; JASCO, Tokyo, Japan).

The bead morphologies were observed by SEM (TM4000Plus-II, Hitachi High-tech, Tokyo, Japan) for each polymer modification step.

### HPLC analysis using the prepared beads packed column

The prepared beads were packed into a stainless-steel column (4.6 mm diameter × 50 mm length). The PNIPAAm-modified beads (0.8 g) were suspended in a methanol/water (1:1) mixture. The suspension was then poured into a column packer connected to a stainless-steel column. Beads were packed by pouring the methanol/water (1:1) suspension into the column at a constant pressure of 35 MPa for 90 min. After packing, pure water was used to rinse the column for 5 h.

The bead-packed column was connected to an HPLC system (Chromaster, Hitachi High-Tech Science, Tokyo, Japan). The column performance was evaluated by HPLC by examining the elution behavior of steroid analytes. The results are summarized in Table S1. A sample of five steroids (50 μg/mL**)** in methanol was prepared. Ten types of drugs required for therapeutic drug monitoring were used as samples. The properties of the drugs are summarized in Table S2. All drugs, except vancomycin, were dissolved in tetrahydrofuran at a concentration of 200 μg/mL. Vancomycin was dissolved in water at a concentration of 200 µg/mL. A CH_3_COONH_4_ solution (pH 6.75 and pH 4.8) was used as the mobile phase. The elution behavior of these analytes was observed at a predetermined wavelength for each drug (Table S2) using a UV detector. A serum drug sample was prepared using the following procedure: Freeze dried serum was purchased from Nissui Pharmaceutical Co. (Tokyo, Japan). Serum was prepared by dissolving freeze-dried serum in water (3 mL). All drugs except vancomycin were dissolved in tetrahydrofuran at a concentration of 1.0 mg/mL. Then, 0.20 mL of the solution was collected in a microtube. The solvent was evaporated using a stream of flowing nitrogen. Then, 1.0 mL of serum solution was added into the microtube, resulting in a 200 μg/mL concentration of drug sample in serum. Vancomycin was dissolved in water at a concentration of 1.0 mg/mL. Then, 0.2 mL of the solution was collected in a microtube, and 0.8 mL of serum solution was added to the microtube, resulting in the drug concentration of 200 μg/mL.

## Supplementary Information

Supplementary Information 1.
