# Supplementary material for: Temperature responsive chromatography for therapeutic drug monitoring with an aqueous mobile phase
Source: Sci Rep. 2021 Dec 6;11:23508. doi: 10.1038/s41598-021-02998-2 (PMC8648775; doi:10.1038/s41598-021-02998-2)
Supplement: Supplementary file 1 — Supplementary Information 1. [file 41598_2021_2998_MOESM1_ESM.docx]

Supplementary Information

**Temperature responsive chromatography for therapeutic drug monitoring with an aqueous mobile phase**

*Kenichi Nagase*, Teruno Nishiyama, Masakazu Inoue, and Hideko Kanazawa*

Faculty of Pharmacy, Keio University, 1-5-30 Shibakoen, Minato, Tokyo 105-8512, Japan

*Corresponding author: (Phone) +81-3-5400-1378; (E-mail) nagase-kn@pha.keio.ac.jp

**Materials**

*N*-Isopropylacrylamide (NIPAAm) was provided by KJ Chemicals (Tokyo, Japan), and it was purified by recrystallization from *n*-hexane. *N,N'* -methylene*bis*acrylamide (BIS), 4,4′-azobis(4-cyanovaleric acid) (V-501), methanol, ethanol, hydrocortisone, prednisolone, dexamethasone, hydrocortisone acetate, testosterone, acetic acid, ammonium acetate, phenytoin, carbamazepine, mycophenolic acid, and vancomycin methotrexate were purchased from Fujifilm Wako Chemicals (Osaka, Japan). *N*-ethoxycarbonyl-2-ethoxy-1,2-dihydroquinoline (EEDQ) was obtained from Peptide Institute (Osaka, Japan). *N*,*N*-dimethylformamide and tetrahydrofuran were obtained from Kanto Chemicals (Tokyo, Japan). Hydrocortisone acetate, lamotrigine, disopyramide, quinidine, propafenone hydrochloride, and digoxin were obtained from Tokyo Chemical Industries (Tokyo, Japan). Freeze-dried serum was obtained from Nissui Pharmaceutical Co. (Tokyo, Japan). Amino propyl silica beads (diameter: 5μm; pore diameter: 120Å; 310 m^2^/g) were purchased from YMC (Kyoto, Japan). Stainless-steel columns (inner diameter: 4.6 mm; column length: 50 mm) were purchased from Nishio Kogyo (Tokyo, Japan).

**Supplementary Table S1.** Properties of hydrophobic steroids

| Compounds | Structure | Molecular weight | Log*P* ^a^ |
| --- | --- | --- | --- |
| Hydrocortisone | 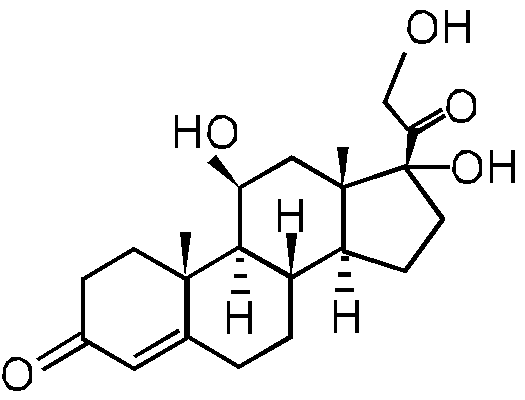 | 362.46 | 1.61 |
| Prednisolone | 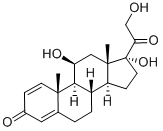 | 360.44 | 1.62 |
| Dexamethasone | 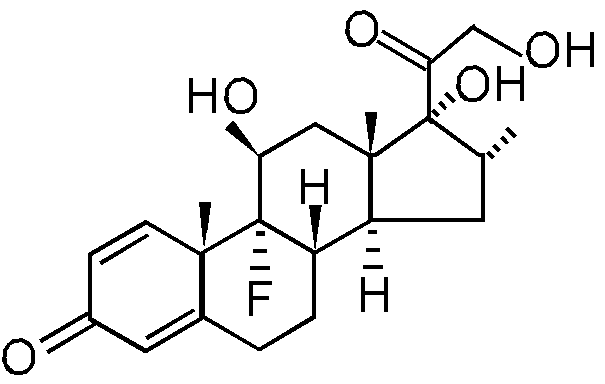 | 392.46 | 1.83 |
| Hydrocortisone acetate | 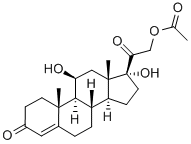 | 404.50 | 2.30 |
| Testosterone | 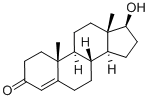 | 288.42 | 3.32 |

a) Partition coefficient in an *n*-octanol/water system

**Supplementary Table S2.1.** Properties of drugs in therapeutic drug monitoring

| Classification | Compounds | Structure | Molecular weight | Log*P* ^a)^ | p*K*_a_ | Solubility ^a)^  (mg/L) | Detection  (nm) |
| --- | --- | --- | --- | --- | --- | --- | --- |
| Antiepileptic drug | Phenytoin | 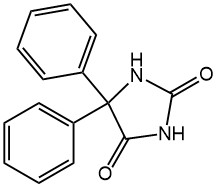 | 252.27 | 2.47 | 8.33 | 32  (at 22°C) | 210 |
|  | Lamotrigine | 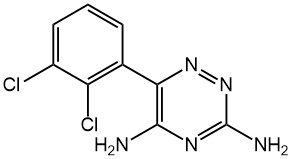 | 256.09 | 2.57 | 5.70 | 170  (at 25°C) | 310 |
|  | Carbamazepine | 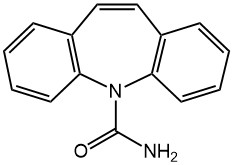 | 236.27 | 2.45 | –3.80, 15.96 | 18  (at 25°C) | 280 |
| Anti-arrhythmic drug | Disopyramide | 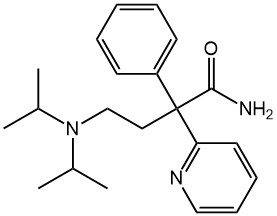 | 339.48 | 2.58 | 10.20 | 44.9 | 260 |
|  | Quinidine | 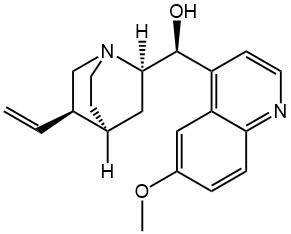 | 324.42 | 3.44 | 8.56 | 140  (at 25°C) | 235 |
|  | Propafenone | 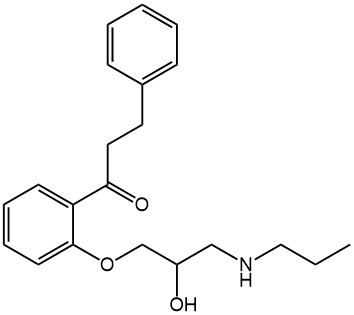 | 341.44 | 3.20 | 8.91 | 93.28  (at 25°C) | 300 |

1. solubility in water.

**Supplementary Table S2.2.** Properties of drugs in therapeutic drug monitoring

| Classification | Compounds | Structure | Molecular weight | Log*P* | p*K*_a_ | Solubility ^a)^  (mg/L) | Detection  (nm) |
| --- | --- | --- | --- | --- | --- | --- | --- |
| Cardiac glycoside | Digoxin | 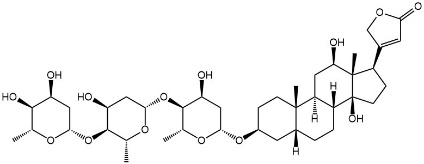 | 780.94 | 1.26 | –3.00, 7.15 | 64.8  (at 25°C) | 220 |
| Anti-bacterial drug | Vancomycin | 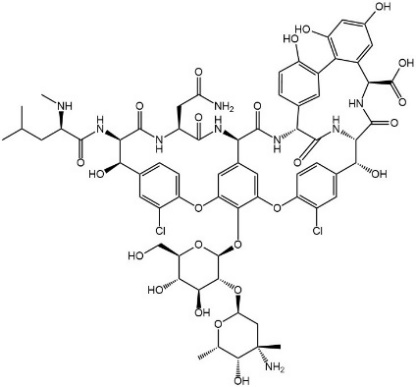 | 1449.27 | -3.10 | 2.60, 7.20, 8.60, 9.60, 10.5, 11.7 | > 100 | 280 |
| Immune-suppressing drug | Mycophenolic acid | 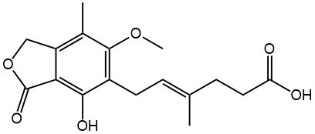 | 320.34 | 2.80 | 5.60 | 10 mM | 300 |
| Anti-cancer drug | Methotrexate | 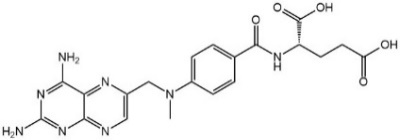 | 454.44 | -1.85 | 4.70 | 29  (at 25°C) | 300 |

1. solubility in water.

**Supplementary Table S3.** Retention time of drugs with repeated measurement

| Drugs | Retention time (min) ^a)^ | RSD (%) ^b)^ |
| --- | --- | --- |
| Phenytoin | 5.78 | 0.05 |
| Lamotrigine | 2.49 | 0.00 |
| Carbamazepine | 4.36 | 0.03 |
| Disopyramide | 1.56 | 0.00 |
| Quinidine | 5.27 | 0.08 |
| Propafenone | 4.75 | 0.04 |
| Mycophenolic acid | 7.81 | 0.11 |
| Digoxin | 1.65 | 0.17 |
| Methotrexate | 7.03 | 0.08 |

a) The retention times of the drugs were measured at 30 °C using 10 mM CH_3_COONH_4_ buffer solution (pH 4.80) as mobile phase and averaged by three separate measurements. b) The relative standard deviation was obtained by dividing the standard deviation of the retention time by the retention time and then centuplicated.


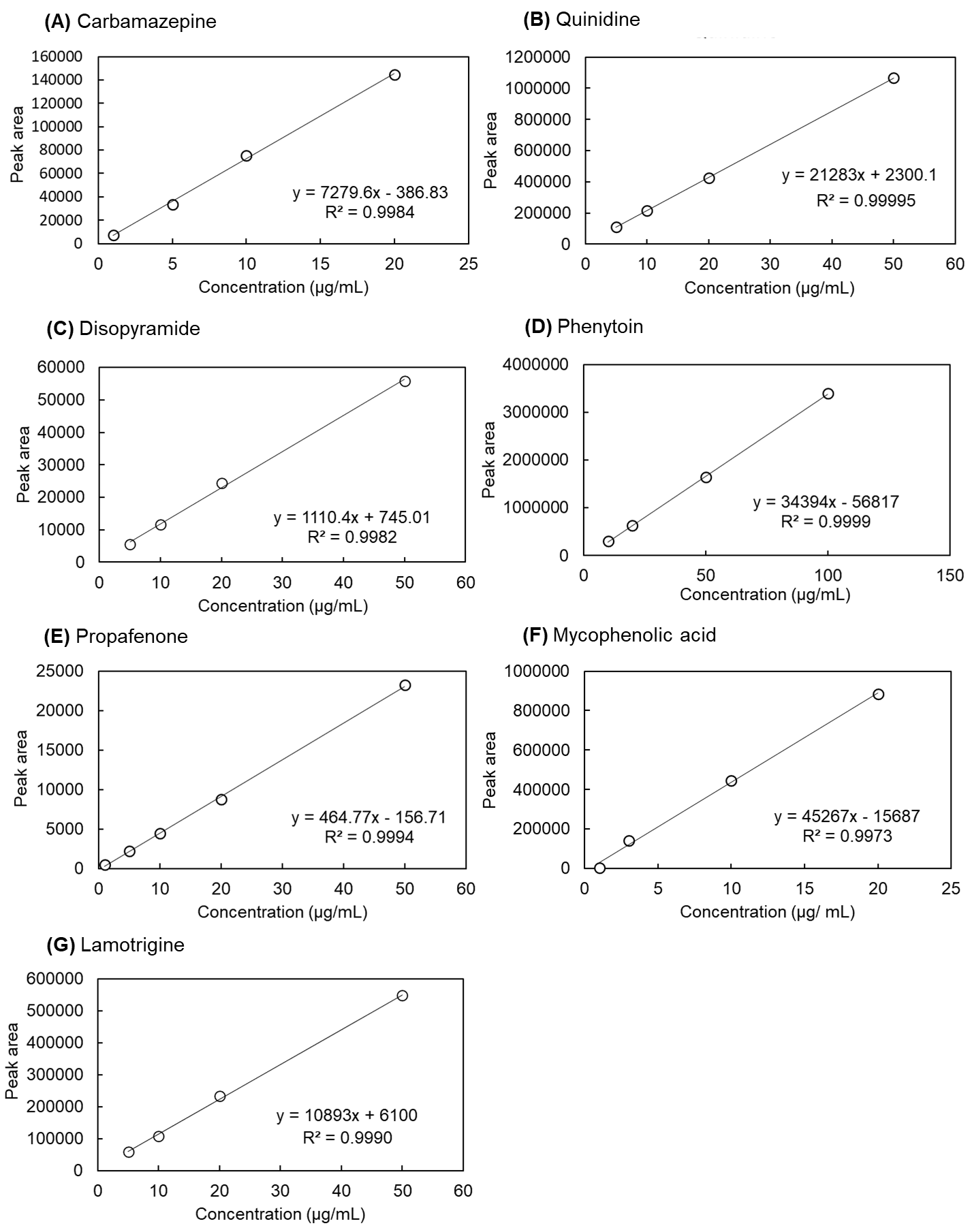


**Supplementary Figure S1.** Calibration curve of drugs used in therapeutic drug monitoring (n = 3)
